# Eu^3+^, Tb^3+^- and Er^3+^, Yb^3+^-Doped α-MoO_3_ Nanosheets for Optical Luminescent Thermometry

**DOI:** 10.3390/nano9040646

**Published:** 2019-04-21

**Authors:** Jing Liu, Rik Van Deun, Anna M. Kaczmarek

**Affiliations:** L3—Luminescent Lanthanide Lab, Department of Chemistry, Ghent University, Krijgslaan 281-S3, B-9000 Ghent, Belgium; jing.liu@ugent.be

**Keywords:** α-MoO_3_:Ln^3+^, a two-step synthesis, temperature sensing

## Abstract

Here we report a novel synthesis approach for the preparation of α-MoO_3_:Ln^3+^ materials employing a two-step synthesis. Additionally, in this work the α-MoO_3_:Ln^3+^ materials are reported as potential optical thermometers for the first time. In this synthesis approach, first MoS_2_ 2D nanosheets were prepared, which were further heat treated to obtain α-MoO_3_. These materials were fully characterized by powder X-ray diffraction (XRD), Raman spectroscopy, X-ray photoelectron spectroscopy (XPS), X-ray fluorescence (XRF), thermogravimetry (TG) and differential thermal analysis (DTA), transmission electron microscopy (TEM), and luminescence spectroscopy. Temperature-dependent luminescence measurements were carried out to determine the optical thermometric properties of two different types of α-MoO_3_:Ln^3+^ materials (Eu^3+^/Tb^3+^ downshifting and Er^3+^/Yb^3+^ upconversion luminescence systems). We demonstrate in this study that this class of material could be a potential candidate for temperature-sensing applications.

## 1. Introduction

Molybdenum trioxide (MoO_3_) is an important transition metal oxide and has two types of crystal phases: Orthorhombic α-MoO_3_ and metastable β-MoO_3_. The physical and chemical properties of these two phases vary, and β-MoO_3_ converts to α-MoO_3_ when heated above 400 °C with a moderate heating rate [1,2]. α-MoO_3_ is the most thermodynamically stable phase with an anisotropic structure with layers parallel to the (010) crystal plane and it consists of bilayered MoO_6_ octahedron corner-sharing chains. It has attracted the most interest due to its unique two-dimensional layered structure [3,4].

Numerous methods have been reported for the synthesis of α-MoO_3_. For example, Afsharpour et al. proposed dissolving amino carboxylates and molybdic acid in an ethanol–water solution, and α-MoO_3_ was obtained by heating the as-prepared nanohybrids at 600 °C [5]. Chen et al. reported mixing MoO_3_·H_2_O with *n*-octylamine at room temperature for 72 h to obtain a white molybdate-based hybrid suspension. After centrifuging the suspension, further washing it with ethanol several times, drying the product for three days, and then placing in the furnace at 550 °C for 1 h, α-MoO_3_ was obtained [6]. Klinbumrung et al. reported that (NH_4_)_6_Mo_7_O_24_·4H_2_O powder could be heated by microwave plasma to decompose the material and obtain α-MoO_3_ [7]. Zhang et al. prepared α-MoO_3_/carbon nanotubes by an electrodeposition method. The electrodeposition solution was first prepared by dissolving molybdenum powder in hydrogen peroxide solution and diluting it with distilled water to the appropriate concentration of molybdenum. Nitrogen-doped carbon nanotubes were used as the electrode, a Pt foil as the counter electrode, and an Ag/AgCl electrode as the reference electrode. The α-MoO_3_ was electrodeposited by a potentiostatic technique. The product was heat treated at different temperatures (300–400 °C) for 2 h in air atmosphere [8]. In this work, we report a novel synthesis approach for the preparation of single-phase α-MoO_3_ material through the oxidation of MoS_2_ sheets. The huge advantage of this method is its simplicity of preparation and the fact that it yields pure single-crystalline α-MoO_3_.

α-MoO_3_ materials have already been considered for many important applications, such as charge storage properties, gas-sensing properties, and photoresponsive properties, but to the best of our knowledge, the α-MoO_3_ material has not yet been studied for its potential use in optical thermometry [6,9,10]. For an ideal optical thermometer, at least two discriminable emission peaks are needed to monitor the signal, and a good optical thermometer should also have high temperature sensitivity [11,12]. It should be pointed out that the calculated thermometry parameters are dependent on the measuring conditions. It has been observed that some errors in the experiments were observed when measurements were carried on different detection systems, and the calculated thermometry parameters differed somewhat under different powers of the excitation light [13].

Optical temperature sensing in the cryogenic region is of great importance for applications in aerospace and energy storage industries [14]. Up to date, few materials have shown good temperature sensing in the cryogenic region, and they show mostly low relative sensitivities (around 1% K^−1^). Only few exceptions of lanthanide metal organic framework (LnMOF) materials have shown high sensitivity in the cryogenic region, such as Tb_0.957_Eu_0.043_(cpda) (H_3_cpda = 5-(4-carboxyphenyl)-2,6-pyridine-dicarboxylic acid) measured from 40 to 300 K, showing relative sensitivity (S_r_) of 16.0% K^−1^ (at 300 K), and [Tb_0.914_Eu_0.086_]_2_(pda)_3_(H_2_O)·2H_2_O measured from 10 to 325 K, showing S_r_ of 5.96% K^−1^ (at 25 K) [15,16]. The highest reported temperature sensitivity for inorganic–organic hybrid materials is up to 4.9% K^−1^ (at 134 K) in a γ-Fe_2_O_3_:Eu^3+^, Tb^3+^ coated with tetraethyl orthosilicate/aminopropyltriethoxysilane (TEOS/APTES) [17]. Compared to metal-oxide frameworks (MOFs), purely inorganic materials are easier to prepare and significantly more stable.

In general, lanthanide luminescence mechanisms can be divided into three types of spectral conversion: Downshifting—the process when one photon with higher frequency is converted into one photon of lower frequency; downconversion—the process where one higher energy photon is transformed into two lower energy photons; and upconversion—the process where at least two lower energy photons are “added up” to give one higher energy photon. In this paper, two different lanthanide pairs (Eu^3+^, Tb^3+^ and Er^3+^, Yb^3+^) have been introduced into the α-MoO_3_ matrix. The α-MoO_3_:Eu^3+^, Tb^3+^ material shows the downshifting luminescence process and the α-MoO_3_:Er^3+^, Yb^3+^ material exhibits an upconversion luminescence process. The temperature-dependent luminescence investigation has been carried out on both of these luminescence systems. The prepared materials were fully characterized employing powder X-ray diffraction (XRD), Raman spectroscopy, X-ray photoelectron spectroscopy (XPS), X-ray fluorescence (XRF), thermogravimetry (TG) and differential thermal analysis (DTA), transmission electron microscopy (TEM), and luminescence spectroscopy.

## 2. Materials and Methods

### 2.1. Materials

Ammonium molybdate tetrahydrate ((NH_4_)_6_Mo_7_O_24_·4H_2_O, >99%, Chem-lab, Belgium), polyethylene glycol 400 (PEG, Mw ~400, laboratory reagent, BDH Chemicals, England), thioacetamide (CH_3_CSNH_2_, >99%, Acros Organics, German), and rare earth chlorides (RE = Eu, Tb, Er, Yb; ≥99.9%, Sigma-Aldrich, China) were used without further purification.

### 2.2. Synthesis of MoS_2_:Ln^3+^

MoS_2_ nanosheets were prepared using a modified previously reported hydrothermal route [18]. For the synthesis of MoS_2_:0.5% Eu^3+^, 4.5% Tb^3+^, 0.1677 g (NH_4_)_6_Mo_7_O_24_·4H_2_O and 0.5 g of PEG were dissolved in 20 mL distilled water and continuously stirred for half an hour. Next, a solution of 2 mmol CH_3_CSNH_2_ with 0.005 mmol EuCl_3_·6H_2_O and 0.045 mmol TbCl_3_·6H_2_O dissolved in 20 mL water was added. The entire mixture was transferred into a stainless-steel autoclave and tightly sealed. The autoclave was heated to 180 °C for 18 h, and afterwards left to cool down to room temperature naturally. The product was separated by centrifugation and washed with ethanol and deionized water several times. Afterwards it was dried in an oven at 50 °C.

For the synthesis of MoS_2_:1% Er^3+^, 10% Yb^3+^, the synthesis was similar to the one discussed above, except for a changing in the lanthanide chloride salts to 0.01 mmol ErCl_3_·6H_2_O and 0.1 mmol YbCl_3_·6H_2_O precursors.

### 2.3. Synthesis of α-MoO_3_

The MoS_2_ nanosheets obtained from the first step were heat treated in air at 600 °C for 3 h to fully oxidize to α-MoO_3_.

### 2.4. Characterization

Powder X-ray diffraction (XRD) patterns were measured on a Thermo Scientific ARL X’TRA diffractometer at a scanning rate of 1.2° min^−1^, at the range of 10–60°. XPS measurements were recorded on an X-ray photoelectron spectroscopy S-Probe XPS spectrometer with monochromatic Al (1486 eV) exciting radiation from Surface Science Instruments (VG). An electron flood gun of 3 eV was used as a neutralizer. All measurements were calibrated towards a value for the C 1s peak of adventitious carbon at 284.6 eV. X-ray fluorescence (XRF) was measured by XRF Supermini200 Rigaku to analyze relative Mo^6+^ and Ln^3+^ contents. Raman spectra were measured by a dispersive Raman spectrometer with a 532 nm laser (Kaiser). Thermogravimetry (TGA) and differential thermal analysis (DTA) were performed on a Stanton Redcroft 1500 apparatus under air flow, with a temperature increase from 20 to 1200 °C at a heating rate of 10 °C/min. The photoluminescence spectra were recorded on an Edinburgh Instruments FLSP 920 UV-vis-NIR spectrofluorimeter with a 450 W xenon lamp for downshifting luminescence or a continuous-wave 975 nm laser for upconversion luminescence as the steady state excitation source. The measurement conditions were as follows, step size = 1.0 nm, ∆λ_em_ = 1.0 nm, ∆λ_ex_ = 1.0 nm, dwell time = 0.2 s. The temperature-dependent measurements were performed using an Advanced Research Systems (ARS) closed-cycle cryostat. All temperature-dependent calculations were performed employing the TeSen software (http://www.tesen.ugent.be) [19]. Transmission electron microscopy (TEM) images were carried out by using a Cs-corrected JEOL JEM2200FS transmission electron microscope with a working voltage of 200 kV.

## 3. Results and Discussion

### 3.1. Structure and Morphology Analysis

**Figure 1:**
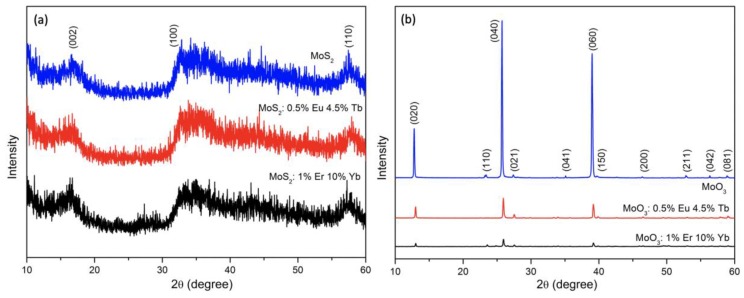
XRD patterns of (**a**) MoS_2_ and (**b**) α-MoO_3_ samples.

The powder XRD patterns of MoS_2_, MoS_2_:Eu, Tb and MoS_2_:Er, Yb could be well matched to the standard JCPDS No. 37-1492. Also the XRD patterns of α-MoO_3_, α-MoO_3_:Eu, Tb, and α-MoO_3_:Er, Yb obtained after heat treating MoS_2_ samples could be well matched to the standard orthorhombic α-MoO_3_ JCPDS No. 05-0508 (presented in Figure 1a,b, respectively). The oxidation of MoS_2_ can be presented with the following chemical reaction: 2MoS_2_ + 7O_2_ → 2MoO_3_ + 4SO_2_. Although no MoS_2_ peaks were found in the XRD patterns of α-MoO_3_ samples, additional characterization techniques were employed to verify a full conversion of MoS_2_ to MoO_3_. Two characteristic peaks in Raman spectra (Figure S1) at around 379 and 405 cm^−1^ were assigned to the E_2g_ and A_1g_ modes of 2H MoS_2_, respectively, where the E_2g_ mode corresponds to the S atoms vibrating in one direction and the M atom vibrating in the other, and A_1g_ mode corresponds to S atoms vibrating out of plane. This further confirmed the successful synthesis of MoS_2_ in the first step reaction [20]. For α-MoO_3_, there are R_c_ modes at 217 cm^−1^, O=Mo=O twisting modes at 245 cm^−1^, O=Mo=O wagging modes at 285 cm^−1^, O–Mo–O bending modes at 337 cm^−1^, O–Mo–O scissoring mode at 366 and 379 cm^−1^, O–Mo–O asymmetric stretching/bending modes at 471 cm^−1^, Mo_3_–O stretching modes at 666 cm^−1^, Mo_2_–O stretching modes at 820 cm^−1^, Mo=O asymmetric stretching modes of terminal oxygen at 995 cm^−1^. These modes are in agreement with the results reported by Klinbumrung [7]. The vibration modes of 2H MoS_2_ could not be found in the Raman spectra of α-MoO_3_ [4,7]. XPS results confirmed the formation of MoS_2_ in the first step of the synthesis, showing that the oxidation state of Mo and S is 4+ and -2, respectively (Figure S2). The composition of MoS_2_ was determined as follows, Mo:S = 36.5:63.5. Compared with Figure S2b,c, a single peak at 162 eV pointing towards a S^2−^ oxidation state in MoS_2_ disappeared in α-MoO_3_, and the peak position of Mo3d_5/2_ at 228.4 eV in MoS_2_ changed to 232.8 eV, indicating Mo^4+^ changed to the Mo^6+^ oxidation state, which proves MoS_2_ fully transformed to α-MoO_3_ [21,22]. The TG and DTA curves of MoS_2_, shown in Figure S3, indicate that MoS_2_ oxidizes to α-MoO_3_ at 556 °C, and after around 750 °C α-MoO_3_ starts to reduce to metallic Mo, which indicates that 600 °C used in the second step of our synthesis is a suitable heat-treatment temperature, and assures obtaining pure α-MoO_3_. The TG and DTA curves of α-MoO_3_ also prove the material is stable in the temperature range from room temperature to around 750 °C, which means the structure of this material will not change in the later temperature-dependent luminescence measurements. XRF results present the real composition of Ln^3+^ in Ln^3+^ co-doped α-MoO_3_ (Table S1), and the Ln^3+^ contents are reasonable compared to the amounts of reagents used in the synthesis.

**Figure 2:**
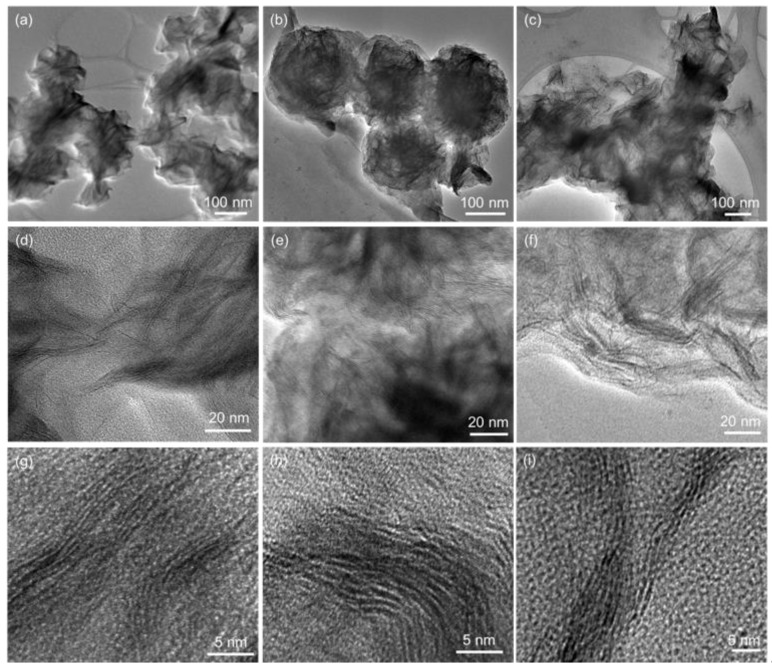
TEM and high-resolution transmission electron microscopy (HRTEM) images of (**a**,**d**,**g**) MoS_2_; (**b**,**e**,**h**) MoS_2_:Eu^3+^, Tb^3+^; (**c**,**f**,**i**) MoS_2_:Er^3+^, Yb^3+^ nanosheets.

**Figure 3:**
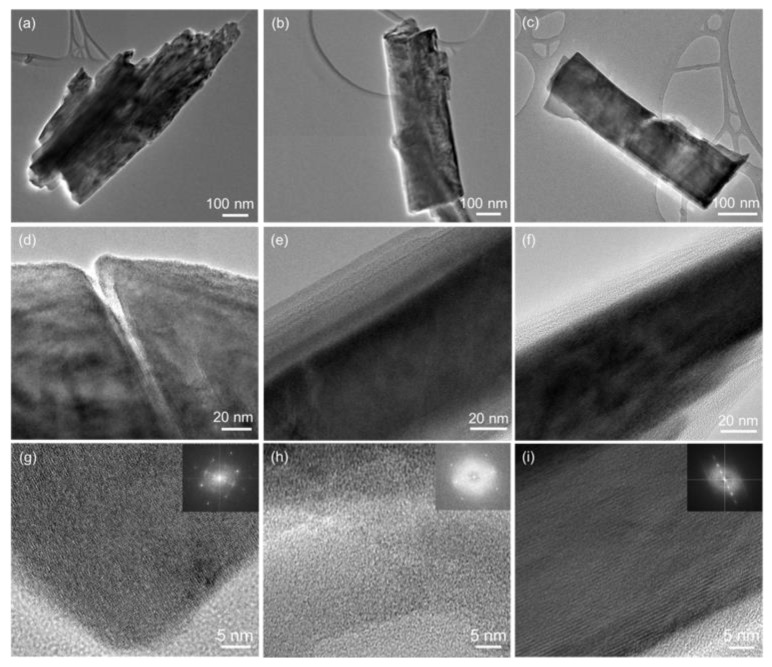
TEM and HRTEM images of (**a**,**d**,**g**) α-MoO_3_; (**b**,**e**,**h**) α-MoO_3_:Eu^3+^, Tb^3+^; (**c**,**f**,**i**) α-MoO_3_:Er^3+^, Yb^3+^ nanosheets.

TEM images presented in Figure 2 depict that MoS_2_ is built up of thin nanosheets, with lots of folds on the surface due to large surface tension. The morphology did not show any obvious changes when Ln^3+^ ions were introduced into the materials. From the high-resolution transmission electron microscopy (HRTEM) images, we did not observe clear lattice fringes, which can be explained by the poor crystallinity of the materials, as observed in the XRD patterns. The structure and morphology of the obtained MoS_2_ are consistent with our previous report [23]. Figure 3 shows the morphology of the α-MoO_3_ material obtained by heat treating MoS_2_ at 600 °C for 3 h in air. In the low magnification TEM images no individual nanoplates were found, but several nanoplates had packed and grown together. The nanoplates are very thin, seen from the shallow contrast grades, which are around 500 nm in length and 130 nm in width. The heat-treatment process helps the nanosheets grow and break down to microsized microplates, but the plates remain thin. From the high-resolution TEM (HRTEM) images, clear lattice fringes were observed, and Fourier transform algorithm (FFT) patterns shown in the insets of Figure 3g–i, confirm it is constituted by a highly ordered diffraction dot array, which suggested α-MoO_3_ has a single-crystal structure [6]. Doping Ln^3+^ did not cause any obvious difference in the morphology of α-MoO_3_.

### 3.2. Luminescence Properties

Luminescence measurements were performed for the α-MoO_3_ materials doped with different percentages of Eu^3+^ ions. As shown in Figures S4 and S5b, at a very low doping percentage (0.25%, 0.5%), a weak broad emission band with a maximum around 439 nm is visible, which can be assigned to the α-MoO_3_ matrix (seen in Figure S6b). The f–f transitions of Eu^3+^ in the emission and excitation spectra have been assigned to appropriate transitions in Table S2. α-MoO_3_:Tb^3+^ materials were also prepared, and the luminescence spectra of 5% Tb^3+^-doped α-MoO_3_ is presented in Figure S7, and the peaks of Tb^3+^ have been assigned to appropriate transitions in Table S3.

**Figure 4:**
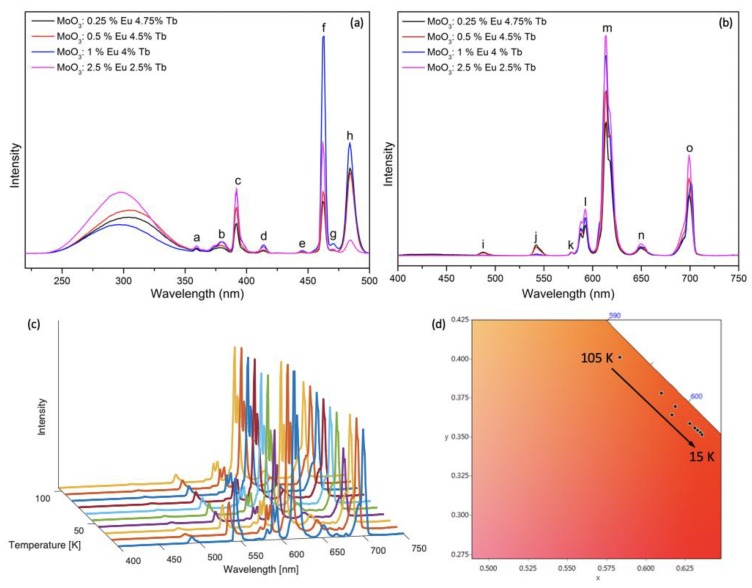
(**a**) Excitation and (**b**) emission spectra of Eu^3+^, Tb^3+^ co-doped α-MoO_3_ at different Eu^3+^/Tb^3+^ percentages, recorded at room temperature (λ_ex_ = 305 nm; λ_em_ = 614 nm); (**c**) the temperature evolution of the emission spectra of α-MoO_3_:0.5% Eu^3+^, 4.5% Tb^3+^ recorded in the range of 15–105 K (step of 10 K); (**d**) Commission Internationale de l’Eclairage (CIE) diagram presenting the observed color change with temperature change.

α-MoO_3_ co-doped materials with different ratios of Eu^3+^ and Tb^3+^ have also been prepared and their luminescence properties have been studied and are presented in Figure 4. The broad band centered round 300 nm in the excitation spectra shown in Figure 4a was ascribed to Mo–O charge transfer band, the other f–f transition peaks of Eu^3+^ and Tb^3+^ have been assigned to appropriate transitions in Table S4. The luminescence lifetimes of the four samples have been included in Table S5. The decreased lifetime of Tb^3+^ emission and increased lifetime of the Eu^3+^ emission indicated the possible energy transfer from Eu^3+^ to Tb^3+^. The temperature-dependent luminescence properties of an α-MoO_3_:0.5% Eu^3+^, 4.5% Tb^3+^ sample were studied and showed interesting behavior in the temperature range of 15–105 K. In the temperature range 15–45 K, we can observe the emission intensity of Tb^3+^ transitions peaks at 488 and 542 nm decreased, and the emission intensity of Eu^3+^ transitions peaks at 592 and 614 nm also decreased, which suggests the multiphoton relaxation is more dominant than the energy transfer process. In the temperature range 45–105 K, the emission intensity at 488 and 542 nm decreased, while the emission intensity at 592 and 614 nm increased, suggesting that multiphoton relaxation is less dominant than the energy transfer process. The probability of multiphoton relaxation will gradually increase with the increase of temperature; and when the temperature is low, the transmission efficiency is greatly affected by temperature, and when the temperature is higher, the probability of multiphoton relaxation is greatly affected by temperature. The corresponding CIE coordinates (Table S6) are indexed in the CIE diagram, which has been presented in Figure 4d.

**Figure 5:**
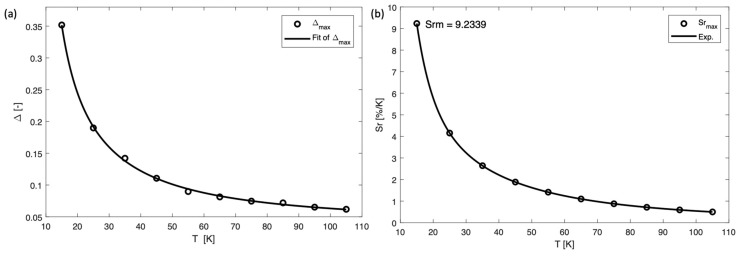
(**a**) Plot presenting the ∆ calculated at different temperatures and (**b**) plot showing the relativity sensitivity (S_r_) at different temperatures.

The thermometric parameter ∆ can be expressed using the following Equations (1)–(3) or (4) [12,24,25]:

(1) $$\Delta \;=\frac{I_{1}}{I_{2}}$$

(2) $$\Delta \;=\;\alpha \exp\left(-\frac{\Delta E}{k_{B}T}\right)$$

(3) $$\Delta \;=\;\frac{\Delta_{0}}{1+\alpha \exp\left(-\frac{\Delta E}{k_{B}T}\right)}$$

(4) $$\Delta \;=\;\frac{\Delta_{0}}{1\;+\;\alpha_{1}\exp\left(-\frac{\Delta E_{1}}{k_{B}T}\right)\;+\;\alpha_{2}\exp\left(-\frac{\Delta E_{2}}{k_{B}T}\right)},$$

where I_1_ and I_2_ are the maximum intensity of peaks at 542 and 614 nm, respectively. ∆_0_ is the thermometric parameter at T = 0 K, and α = W_0_/W_R_, is the ratio between the nonradiative rates (W_0_ is at T = 0 K) and radiative rates (W_R_). K_B_ is the Boltzmann constant (0.695 cm^−1^ K^−1^), T is the absolute temperature (K), and ∆E is the activation energy of a nonradiative process. For dual-center thermometers, the most commonly used equation is Equation (3), but in this material the overlapping of Tb^3+^ peak and Mo–O charge transfer band of the α-MoO_3_ matrix at around 488 nm caused a possible second nonradiative process, so Equation (4) was employed to calculate the relationship between ∆ and T. Additionally Equation (4) gave a better fit (R^2^ = 0.999) than Equation (3) (R^2^ = 0.996) as shown in Figure 5a. The parameters were fitted to be ∆_0_ = 3.38, α_1_ = 60.20, ∆E_1_ = 70.20 cm^−1^, α_2_ = 38.36, ∆E_2_ = 15.66 cm^−1^. These values were calculated considering the maxima of the 542 and 614 nm peaks. The larger nonradiative deactivation energy (∆E_1_ = 70.20 cm^−1^) probably includes the α-MoO_3_ Mo–O charge transfer band and the ^5^D_4_ level of Tb^3+^, and the smaller nonradiative deactivation energy (∆E_2_ = 15.66 cm^−1^) most likely includes Tb^3+^–Tb^3+^ energy migration.

The absolute sensitivity S_a_ and relative sensitivity S_r_ can be expressed using Equations (5) and (6) [26,27]:

(5) $$S_{a}=\left|\frac{\partial \Delta }{\partial T}\right|$$

(6) $$S_{r}=100\%\times \left|\frac{1}{\Delta }\frac{\partial \Delta }{\partial T}\right|.$$

**Figure 6:**
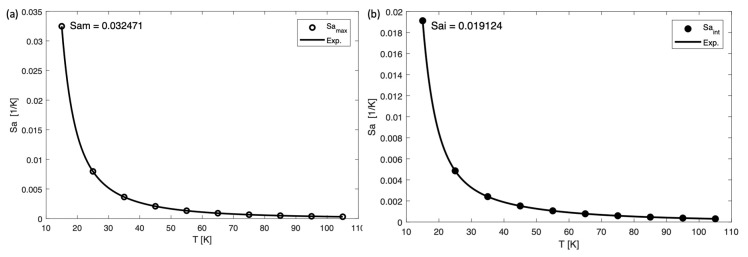
Plot showing the absolute sensitivity (S_a_) of α-MoO_3_:Eu^3+^, Tb^3+^ at different temperatures (**a**) calculated using the maximum intensity (S_am_) of peaks at 512 nm of Tb^3+^ and 614 nm of Eu^3+^; (**b**) calculated using the integrated areas under the peak (S_ai_) from 535 to 554 nm for Tb^3+^ and from 605 to 634 nm for Eu^3+^.

S_r_ is a more important parameter compared to S_a_ for quantifying and comparing the sensitivity of different thermometers as S_a_ is strongly depending on the samples features and the experimental device [28]. Figure 5b and Figure 6a show that the sensitivity decreased continuously, and the maximum value of S_r_ and S_a_ is 9.234% K^−1^ (at 15 K), and 0.0325 K^−1^ (at 15 K), respectively, where S_r_ is a very high value compared to other cryogenic temperature sensors reported in literature (see Table 1). Additionally, the S_a_ and S_r_ values calculated when considering the integrated area under the peaks (from 535 to 554 nm for Tbs^3+^ and from 605 to 634 nm for Eu^3+^) are presented in Figure S8b and Figure 6b, showing S_r_ = 7.621% K^−1^ (at 15 K), and S_a_ = 0.0191 K^−1^ (at 15 K), with parameters ∆_0_ = 3.40, α_1_ = 61.75, ∆E_1_ = 88.78 cm^−1^, α_2_ = 42.55, ∆E_2_ = 12.80 cm^−1^. The calculation based on the peak maxima resulted in a slightly better fit (R^2^ = 0.999) compared to the integrated areas under the peaks (R^2^ = 0.998).

**Figure 7:**
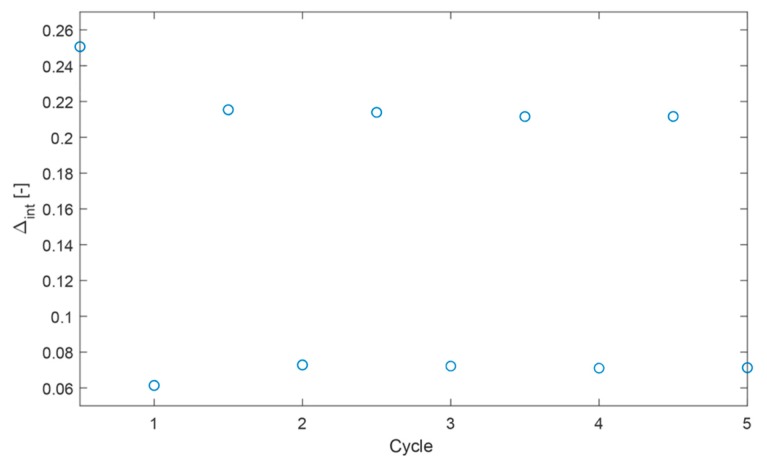
Plot showing temperature-cycle measurements for α-MoO_3_:Eu^3+^, Tb^3+^.

The repeatability (R) of a thermometer indicates the precision of the measurement system, which can be quantified by using Equation (7) [28]:

(7) $$R=1-\frac{max\left|\Delta c\;-\;\Delta i\right|}{\Delta c},$$

where ∆c is the mean thermometric parameter and ∆i is the value of each measurement of thermometric parameter. Temperature-cycle tests (Figure 7) were performed to verify α-MoO_3_:Eu^3+^, Tb^3+^ materials showed good repeatability (R = 92%).

**Figure 8:**
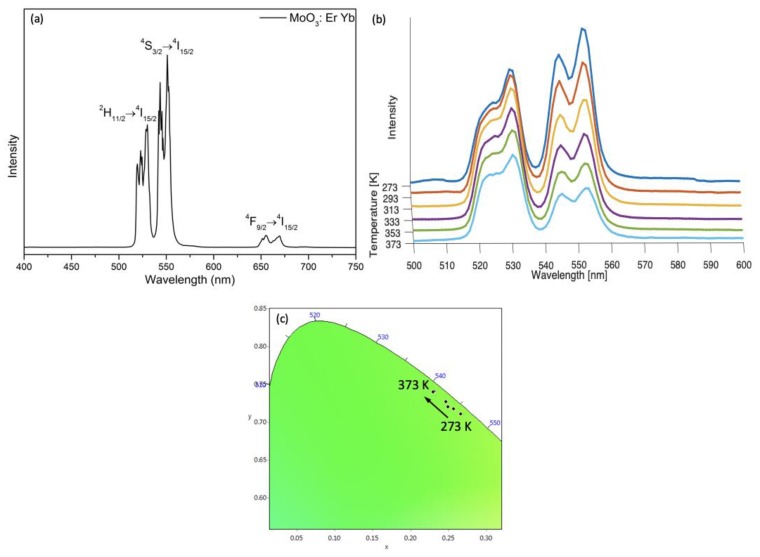
(**a**) Emission spectrum of α-MoO_3_:Er^3+^, Yb^3+^ under excitation at 975 nm measured at room temperature; (**b**) the temperature evolution of the emission spectra of α-MoO_3_:Er^3+^, Yb^3+^ recorded in the range of 273–373 K (step of 20 K); (**c**) CIE diagram presenting the observed color change with temperature change.

**Figure 9:**
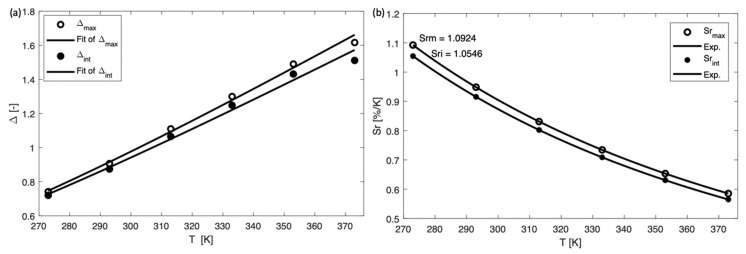
Plot presenting (**a**) the ∆ and (**b**) the relativity sensitivity (S_r_) of α-MoO_3_: 1% Er^3+^, 10% Yb^3+^ calculated at different temperatures.

**Figure 10:**
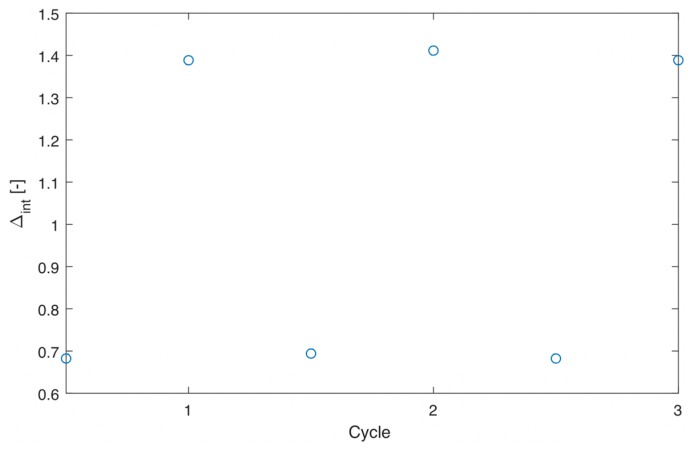
Plot showing temperature-cycle measurements for α-MoO_3_:Er^3+^, Yb^3+^.

Temperature-dependent upconversion luminescence measurements were also performed on the α-MoO_3_:Er^3+^, Yb^3+^ sample. The α-MoO_3_:Er^3+^, Yb^3+^ material showed temperature-sensing behavior in the high-temperature range, but not in the cryogenic range. The room temperature emission spectrum and the temperature evolution of the emission spectra in the temperature range of 273–373 K are shown in Figure 8a,b, respectively. The intensity of two emission peaks decreased with the increase of temperature, since the increase of temperature enhances the probability of nonradiative transition, leading to the luminescence quenching. The color did not change in this particular temperature range, and the CIE coordinates shown in Table S7 are indexed to a CIE diagram in Figure 8c. The maximum intensity of peaks at 530 nm (^2^H_11/2_ *→* ^2^H_15/2_ transition) and 552 nm (^4^S_3/2_ *→* ^4^I_15/2_ transition) of Er^3+^ were used to calculate ∆. The data could be well fitted with Equation (2) for single-center thermometers (Figure 9a, R^2^ = 0.994), yielding parameters α = 14.74, ∆E = 565.86 cm^−1^, which is close to the theoretical calculated value (energy difference between ^2^H_11/2_ and ^4^S_3/2_: 727 cm^−1^). Figure 9b and Figure S10 show S_r_ and S_a_ as a function of temperature, respectively. The maximum value of S_r_ is 1.092% K^−1^ (at 273 K), and S_a_ is 0.00972 K^−1^ (at 273 K). Since the monitored peaks of Er^3+^ overlapped at 538 nm, when ∆ was determined considering the integrated area under the peaks a worse R^2^ value of 0.987 was obtained. Temperature-cycle tests are shown in Figure 10 proving that the material gives excellent repeatability (R = 98%).

## 4. Conclusions

In conclusion, single-crystalline α-MoO_3_ has been successfully synthesized via a novel two-step synthesis based on the heat treatment of pre-synthesized MoS_2_ sheets. Two different pairs of Ln^3+^ ions (Eu, Tb and Er, Yb) were introduced into the α-MoO_3_ matrix to obtain either downshifting or upconversion luminescence. α-MoO_3_ co-doped with Eu^3+^, Tb^3+^ and Er^3+^, Yb^3+^ both showed interesting thermometric properties. The α-MoO_3_:Eu^3+^, Tb^3+^ material showed superior optical sensing properties in the cryogenic region compared to previously reported inorganic materials, reaching a remarkably high maximum relative sensitivity (S_r_) value of 9.234% K^−1^ (at 15 K). α-MoO_3_:Er^3+^, Yb^3+^ showed optical sensing properties in the temperature range of 273–373 K (physiological range), reaching an S_r_ value of 1.092% K^−1^ (at 273 K). These observations are very interesting and in our future work we aim at understanding the mechanism behind the very high thermometric values for the MoO_3_:Eu^3+^, Tb^3+^ material.

## Figures and Tables

**Table 1:** Summary of the maximum relative sensitivity (S_r_) for reported ratiometric thermometers based on lanthanide inorganic materials operating in the cryogenic region.

Material	Temperature Range (K)	Maximum S_r_ (% K^−1^)	Temperature of Maximum S_r_ (K)	Reference
MoS_2_:Eu	60–360	1.49	180	[23]
La_2_O_2_S:Nd	30–600	1.10	358	[29]
Na_0.82_Ca_0.08_YF_4_:Er	5–300	0.22	338	[30]
Y_2_O_3_:Er	93–613	0.44	427	[31]
Y_2_O_3_:Ho, Yb	10–300	0.97	85	[32]
γ-Fe_2_O_3_:Eu, Tb@TEOS/APTES	10–350	4.70	134	[17]
MoO_3_:Eu, Tb	15–105	9.23	15	This work

## Supplementary Materials

The following are available online at https://www.mdpi.com/2079-4991/9/4/646/s1. Figure S1. Raman spectra of (a) MoS_2_ and (b) α-MoO_3_ samples, Figure S2. (a) XPS spectra of MoS_2_ (red) and α-MoO_3_ (green); peak deconvolution in Mo3d, S2s region in (b) MoS_2_ and (c) α-MoO_3_, Figure S3. TG and DTA curves of (a) MoS_2_ and (b) α-MoO_3_ samples, Figure S4. Emission spectra of α-MoO_3_:Eu^3+^ samples (at different Eu^3+^ doping percentages) recorded at room temperature (λex = 283 nm; λem = 614 nm), Figure S5. (a) Excitation spectrum and (b) emission spectrum of α-MoO_3_:0.5%Eu^3+^ recorded at room temperature (λex = 283 nm; λem = 614 nm), Figure S6. (a) Excitation spectrum and (b) emission spectrum of α-MoO_3_ recorded at room temperature (λex = 374 nm; λem = 439 nm), Figure S7. (a) Excitation spectrum and (b) emission spectrum of α-MoO_3_:5%Tb^3+^ recorded at room temperature (λex = 310 nm; λem = 542 nm), Figure S8. (a) Plot presenting the ∆ and (b) the relativity sensitivity (Sr) of α-MoO_3_:Eu^3+^, Tb^3+^ at different temperatures calculated using the integrated areas under the peak (from 535–554 nm for Tb^3+^ and 605–634 nm for Eu^3+^), Figure S9. Decay curve of 551 nm emission of α-MoO_3_:Er^3+^, Yb^3+^ under excitation of 975 nm, Figure S10. Plot showing the absolute sensitivity (Sa) of α-MoO_3_:Er^3+^, Yb^3+^ at different temperatures, Table S1. Relative Mo^6+^ and Ln^3+^ contents (mol %) for α-MoO_3_:Ln^3+^ co-doped samples from synthesis (calcd.) and as determined by XRF, Table S2. Assignment of labeled transition peaks shown in Figure S5, Table S3. Assignment of labeled transition peaks shown in Figure S7, Table S4. Assignment of labeled transition peaks shown in Figure 4a,b, Table S5. Luminescence decay time of α-MoO_3_:Eu^3+^, Tb^3+^ under excitation of 305 nm, Table S6. CIE colour coordinates (x, y) of α-MoO_3_:0.5% Eu^3+^, 4.5% Tb^3+^ calculated at different temperatures, Table S7. CIE colour coordinates (x, y) of α-MoO_3_:1% Er^3+^, 10% Yb^3+^ calculated at different temperatures.
