# Supplementary material for: Eu3+, Tb3+- and Er3+, Yb3+-Doped α-MoO3 Nanosheets for Optical Luminescent Thermometry
Source: Nanomaterials (Basel). 2019 Apr 21;9(4):646. doi: 10.3390/nano9040646 (PMC6524054; doi:10.3390/nano9040646)
Supplement: Supplementary file 1 [file nanomaterials-09-00646-s001.pdf]

Supporting information

Eu^{3+}, Tb^{3+} and Er^{3+}, Yb^{3+} doped α-MoO_3 nanosheets for optical luminescent thermometry

Jing Liu [a], Rik Van Deun [a]* and Anna M. Kaczmarek [a]*

[a] L³ – Luminescent Lanthanide Lab, Department of Chemistry, Ghent University, Krijgslaan 281-S3, B-9000 Ghent, Belgium; corresponding email: Rik.VanDeun@UGent.be; Anna.Kaczmarek@UGent.be

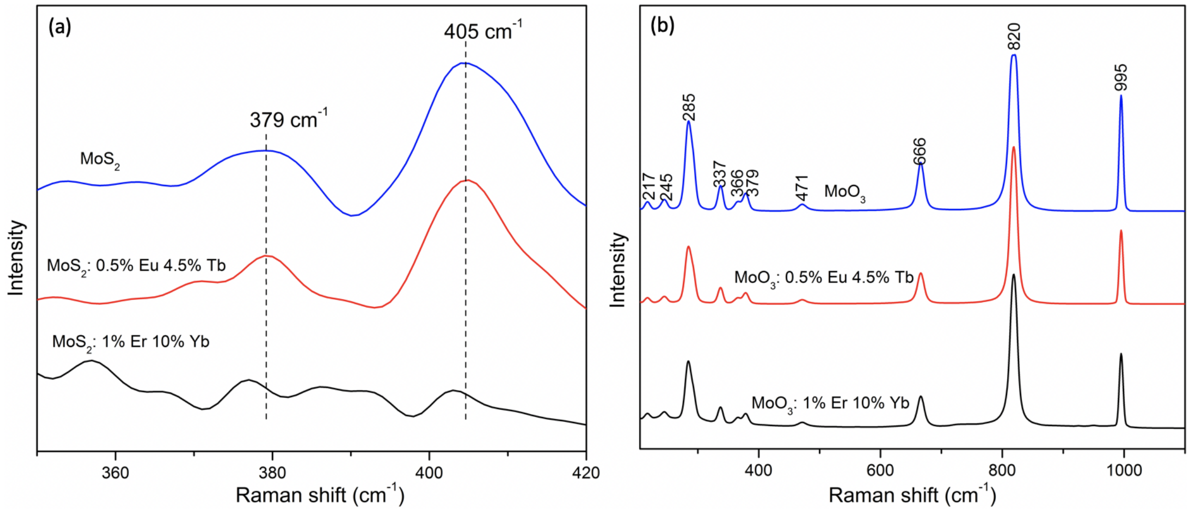

Figure S1. Raman spectra of (a) MoS_2 and (b) α-MoO_3 samples.

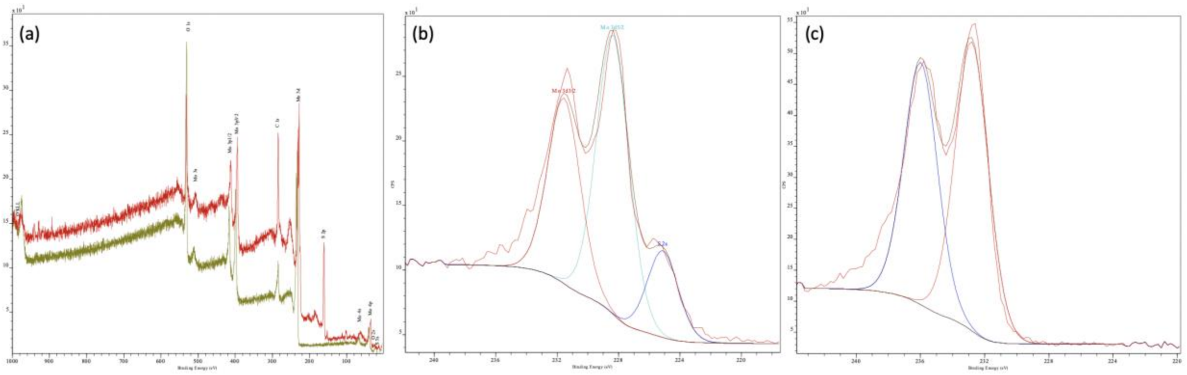

Figure S2. (a) XPS spectra of MoS_2 (red) and α-MoO_3 (green); peak deconvolution in Mo3d, S2s region in (b) MoS_2 and (c) α-MoO_3.

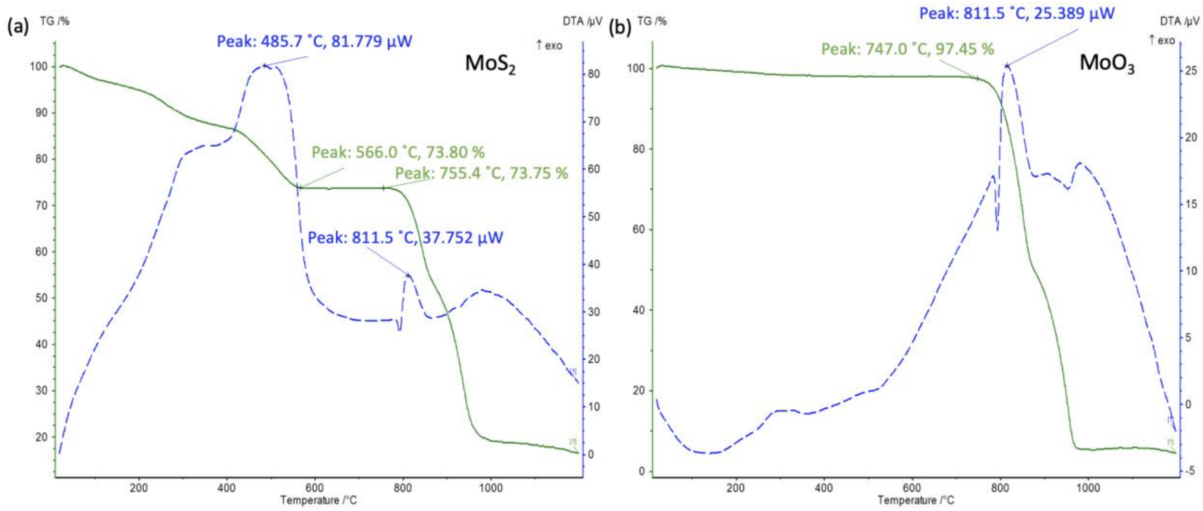

Figure S3. TG and DTA curves of (a) MoS_2 and (b) α-MoO_3 samples.

Table S1. Relative Mo^{6+} and Ln^{3+} contents (mol %) for α-MoO_3:Ln^{3+} co-doped samples from synthesis (calcd.) and as determined by XRF.

Sample	Mo^{6+} (%)		Er^{3+} (%)		Yb^{3+} (%)		Eu^{3+} (%)		Tb^{3+} (%)	
	Calc.	XRF	Calcd.	XRF	Calcd.	XRF	Calcd.	XRF	Calcd.	XRF
α-MoO_3:Er, Yb	89	89.39	1	0.85	10	9.76				
α-MoO_3:Eu, Tb	95	96.87					0.5	0.23	4.5	2.9

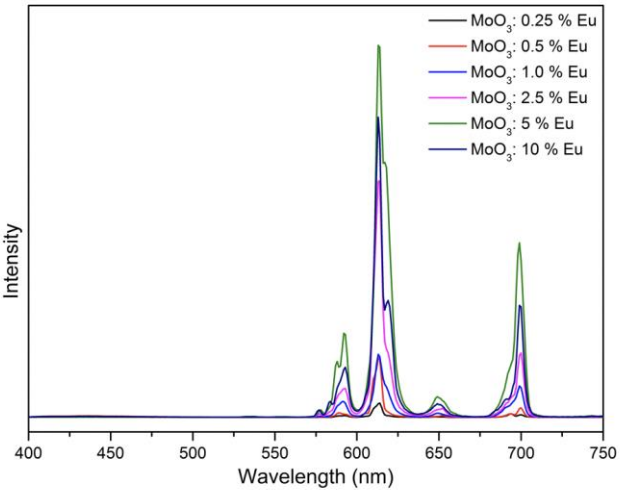

Figure S4. Emission spectra of α-MoO_3:Eu^{3+} samples (at different Eu^{3+} doping percentages) recorded at room temperature (λ_{ex} = 283 nm; λ_{em} = 614 nm).

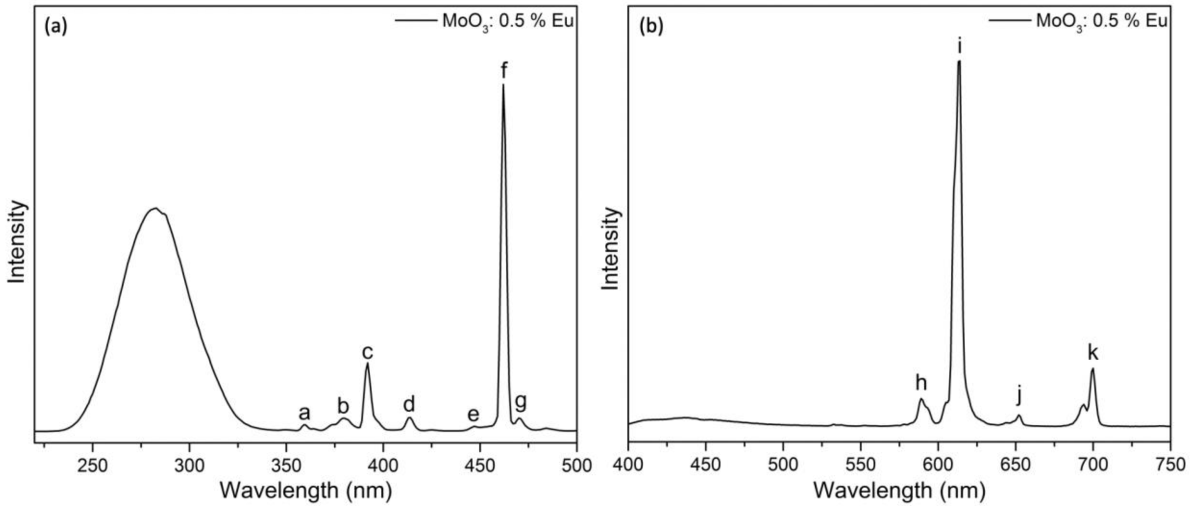

Figure S5. (a) Excitation spectrum and (b) emission spectrum of α-MoO_3:0.5%Eu^{3+} recorded at room temperature (λ_{ex} = 283 nm; λ_{em} = 614 nm).

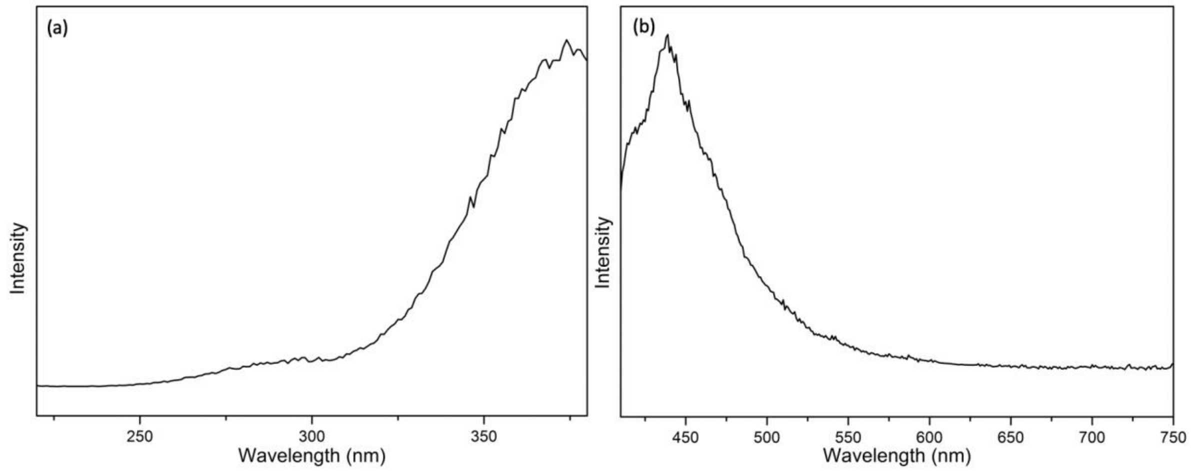

Figure S6. (a) Excitation spectrum and (b) emission spectrum of α-MoO_3 recorded at room temperature (λ_{ex} = 374 nm; λ_{em} = 439 nm).

Table S2. Assignment of labeled transition peaks shown in Figure S5.

	Excitation				Emission		
	Wavelength (nm)	Energy (cm^{-1})	Transition		Wavelength (nm)	Energy (cm^{-1})	Transition
a	359	27855	$^5D_4 \leftarrow {}^7F_0$	h	589	16978	$^5D_0 \rightarrow {}^7F_1$
b	379	26385	$^5G_2 \leftarrow {}^7F_0$	i	614	16287	$^5D_0 \rightarrow {}^7F_2$
c	392	25510	$^5L_6 \leftarrow {}^7F_0$	j	652	15337	$^5D_0 \rightarrow {}^7F_3$
d	414	24154	$^5D_3 \leftarrow {}^7F_1$	k	700	14286	$^5D_0 \rightarrow {}^7F_4$
e	447	22371	$^5D_3 \leftarrow {}^7F_3$				
f	462	21645	$^5D_2 \leftarrow {}^7F_0$				
g	470	21276	$^5D_2 \leftarrow {}^7F_1$				

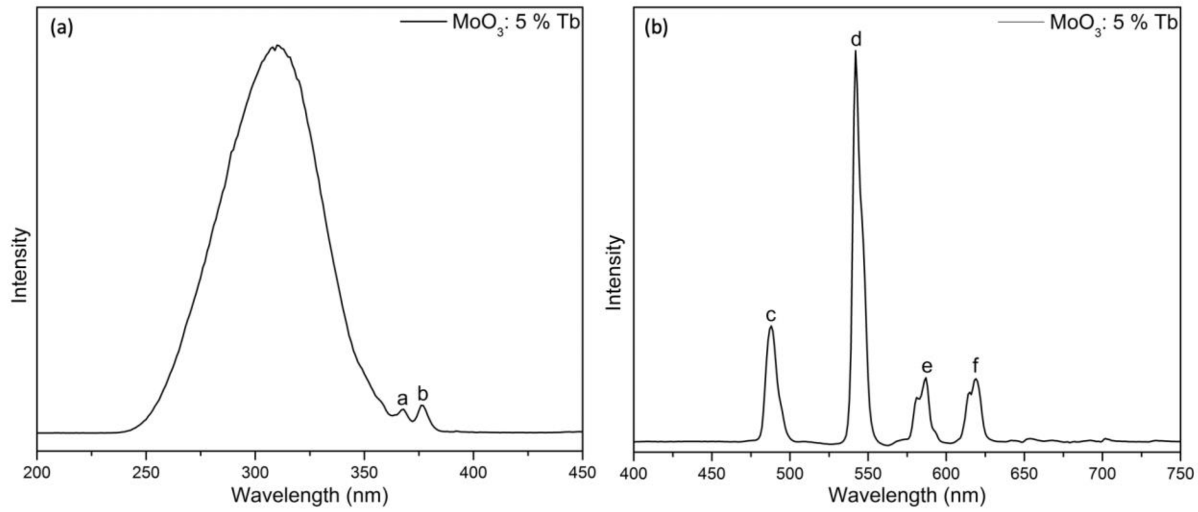

Figure S7. (a) Excitation spectrum and (b) emission spectrum of α-MoO_3:5%Tb^{3+} recorded at room temperature (λ_{ex} = 310 nm; λ_{em} = 542 nm).

Table S3. Assignment of labeled transition peaks shown in Figure S7.

	Excitation				Emission		
	Wavelength (nm)	Energy (cm^{-1})	Transition		Wavelength (nm)	Energy (cm^{-1})	Transition
a	368	27174	$^5L_{10} \leftarrow {}^7F_6$	c	488	20492	$^5D_4 \rightarrow {}^7F_6$
b	376	26596	$^5G_6 \leftarrow {}^7F_6$	d	542	18450	$^5D_4 \rightarrow {}^7F_5$
				e	587	17036	$^5D_4 \rightarrow {}^7F_4$
				f	619	16155	$^5D_4 \rightarrow {}^7F_3$

Table S4. Assignment of labeled transition peaks shown in Figure 4a and 4b.

Excitation					Emission				
	Wavelength (nm)	Energy (cm^{-1})	Eu^{3+} Transition	Tb^{3+} Transition		Wavelength (nm)	Energy (cm^{-1})	Eu^{3+} Transition	Tb^{3+} Transition
a	360	27778	$^5D_4 \leftarrow {}^7F_0$		i	488	20492		$^5D_4 \rightarrow {}^7F_6$
b	379	26385	$^5G_2 \leftarrow {}^7F_0$		j	542	18450		$^5D_4 \rightarrow {}^7F_5$
c	392	25510	$^5L_6 \leftarrow {}^7F_0$		k	578	17301	$^5D_0 \rightarrow {}^7F_0$	$^5D_4 \rightarrow {}^7F_4$
d	414	24154	$^5D_3 \leftarrow {}^7F_1$		l	592	16892	$^5D_0 \rightarrow {}^7F_1$	
e	445	22472	$^5D_3 \leftarrow {}^7F_3$		m	614	16287	$^5D_0 \rightarrow {}^7F_2$	
f	463	21598	$^5D_2 \leftarrow {}^7F_0$		n	650	15385	$^5D_0 \rightarrow {}^7F_3$	
g	470	21276	$^5D_2 \leftarrow {}^7F_1$		o	699	14306	$^5D_0 \rightarrow {}^7F_4$	
h	484	20661		$^5D_4 \leftarrow {}^7F_6$					

Table S5. Luminescence decay time of α-MoO_3:Eu^{3+}, Tb^{3+} under excitation of 305 nm.

Sample name	Decay time (λ_{em} = 542 nm)	Decay time (λ_{em} = 612 nm)
α-MoO_3:0.25% Eu^{3+}, 4.75% Tb^{3+}	101 μs	661 μs
α-MoO_3:0.5% Eu^{3+}, 4.5% Tb^{3+}	70 μs	689 μs
α-MoO_3:1% Eu^{3+}, 4% Tb^{3+}	22 μs	930 μs
α-MoO_3:2.5% Eu^{3+}, 2.5% Tb^{3+}	7 μs	942 μs

Table S6. CIE colour coordinates (x, y) of α-MoO_3:0.5% Eu^{3+}, 4.5% Tb^{3+} calculated at different temperatures.

Temperature (K)	x coordinate	y coordinate
15	0.5835	0.4011
25	0.6099	0.3781
35	0.6188	0.3693
45	0.6168	0.3640
55	0.6281	0.3586
65	0.6313	0.3558
75	0.6331	0.3543
85	0.6347	0.3528
95	0.6360	0.3514
105	0.6370	0.3500

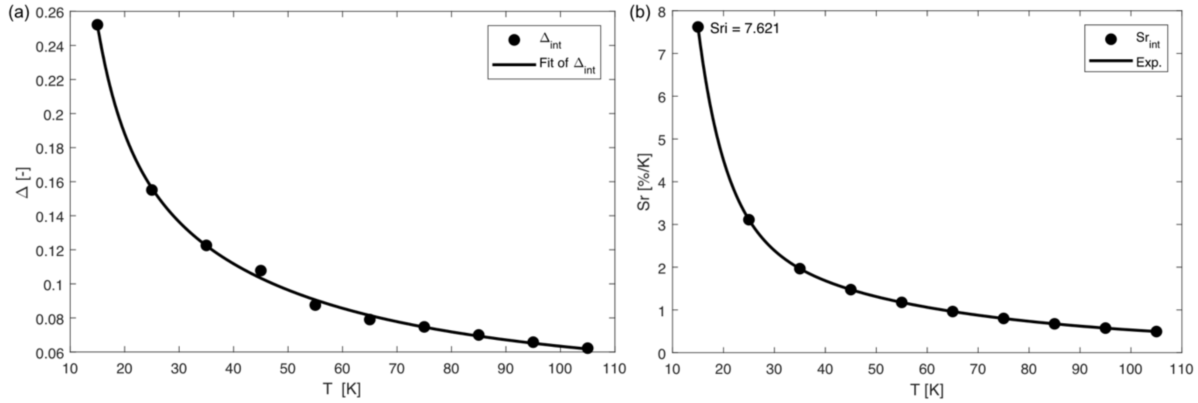

Figure S8. (a) Plot presenting the Δ and (b) the relativity sensitivity (S_r) of α-MoO_3:Eu^{3+}, Tb^{3+} at different temperatures calculated using the integrated areas under the peak (from 535-554 nm for Tb^{3+} and 605-634 nm for Eu^{3+}).

Table S7. CIE colour coordinates (x, y) of α-MoO_3:1% Er^{3+}, 10% Yb^{3+} calculated at different temperatures.

Temperature (K)	x coordinate	y coordinate
273	0.2661	0.7112
293	0.2572	0.7178
313	0.2467	0.7269
333	0.2311	0.7407
353	0.2302	0.7398
373	0.2493	0.7207

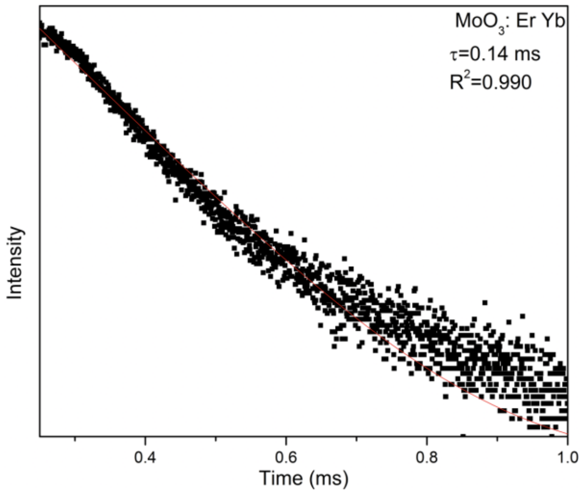

Figure S9. Decay curve of 551 nm emission of α-MoO_3:Er^{3+}, Yb^{3+} under excitation of 975 nm.

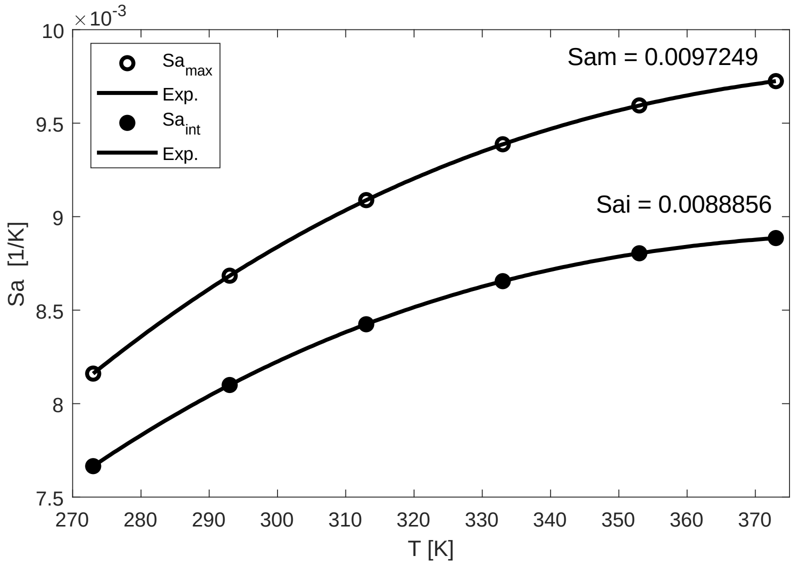

Figure S10. Plot showing the absolute sensitivity (S_a) of α-MoO_3:Er^{3+}, Yb^{3+} at different temperatures.
